# A Look into Liver Mitochondrial Dysfunction as a Hallmark in Progression of Brain Energy Crisis and Development of Neurologic Symptoms in Hepatic Encephalopathy

**DOI:** 10.3390/jcm9072259

**Published:** 2020-07-16

**Authors:** Elena Kosenko, Lyudmila Tikhonova, Gubidat Alilova, Carmina Montoliu

**Affiliations:** 1 Institute of Theoretical and Experimental Biophysics of Russian Academy of Sciences, 142290 Pushchino, Russia; ljudasik09@rambler.ru (L.T.); hells2012@yandex.ru (G.A.); 2 Hospital Clinico Research Foundation, INCLIVA Health Research Institute, 46010 Valencia, Spain; carmina.montoliu@uv.es; 3 Pathology Department, Faculty of Medicine, University of Valencia, 46010 Valencia, Spain

**Keywords:** hyperammonemia, liver, mitochondria, ketogenesis, gluconeogenesis, brain energy crisis

## Abstract

Background: The relationship between liver disease and neuropathology in hepatic encephalopathy is well known, but the genesis of encephalopathy in liver failure is yet to be elucidated. Conceptually, the main cause of hepatic encephalopathy is the accumulation of brain ammonia due to impaired liver detoxification function or occurrence of portosystemic shunt. Yet, as well as taking up toxic ammonia, the liver also produces vital metabolites that ensure normal cerebral function. Given this, for insight into how perturbations in the metabolic capacity of the liver may be related to brain pathology, it is crucial to understand the extent of ammonia-related changes in the hepatic metabolism that provides respiratory fuel for the brain, a deficiency of which can give rise to encephalopathy. Methods: Hepatic encephalopathy was induced in starved rats by injection of ammonium acetate. Ammonia-induced toxicity was evaluated by plasma and freeze-clamped liver and brain energy metabolites, and mitochondrial, cytoplasmic, and microsomal gluconeogenic enzymes, including mitochondrial ketogenic enzymes. Parameters of oxidative phosphorylation were recorded polarographically with a Clark-type electrode, while other measures were determined with standard fluorometric enzymatic methods. Results: Progressive impairment of liver mitochondrial respiration in the initial stage of ammonia-induced hepatotoxicity and the subsequent energy crisis due to decreased ATP synthesis lead to cessation of gluconeogenesis and ketogenesis. Reduction in glucose and ketone body supply to the brain is a terminal event in liver toxicity, preceding the development of coma. Conclusions: Our study provides a framework to further explore the relationship between hepatic dysfunction and progression of brain energy crisis in hepatic encephalopathy.

## 1. Introduction

Hepatic encephalopathy (HE) is a neuropsychiatric disorder developing in patients with severe liver disease. Although the relationship between liver damage and neuropathology has been known for over a century [1,2], the genesis of encephalopathy as a result of impaired liver function is not yet completely clear.

Conceptually, the main cause of HE is the accumulation of ammonia in the blood (hyperammonemia) due to impaired detoxification function of the liver or by portosystemic shunt, allowing gut-derived neurotoxins that bypass the liver to reach the systemic circulation, thereby gaining access to the brain [3].

Other factors such as bacteremia, fungal infection [4], alkalosis [5], products of abnormal amino acid metabolism [6], and various toxic substances [7] may participate in disease development, but elevated blood ammonia entering the brain unhindered is believed to be the main toxic causative agent responsible for altered cerebral functions and clinical manifestations of HE [3,6].

The molecular mechanisms of encephalopathy in the setting of hyperammonemia have not yet been fully elucidated and there is no specific medication for treating this abnormality, despite considerable knowledge of the syndrome.

It has been established that ammonia-related neurotoxicity is characterized by brain energy failure [8], disruption of mitochondrial oxidative phosphorylation [9] and calcium homeostasis [10], an imbalance among multiple neurotransmitter systems [11,12], disturbances of antioxidant defense systems [13,14], systemic neuroinflammation [15], and abnormalities in the pattern of cerebral blood flow [16]. Therefore, HE pathogenesis is viewed as a complex and multifactorial network of interdependent organ systems.

Thus, the impact on functional brain activity as a result of liver dysfunction may be wider than predicted based on increased blood ammonia levels due to the inability of the liver to perform its detoxification function. This hypothesis prompted us to investigate an alternative explanation for the effect of liver malfunction on brain pathology.

It is known that the liver not only takes up toxic ammonia, but also plays a unique role in blood glucose homeostasis [17]. Given that glucose is the main brain energy metabolite, despite the recognized importance of some non-glucose substrates [18], the liver is essential in homeostatic control of blood glucose levels. This process maintains adequate amounts of glucose for the brain and reflects its obligate dependence on the liver, indicating that under physiological conditions, the liver elaborates vital metabolites that assure normal cerebral function [19]. As a result, unsteady blood glucose and oxygen delivery to the brain for even a short period causes brain damage, while chronic deficiency in these substrates leads to irreversible brain injury, thereby provoking coma development and death [20].

Owing to the large stores of glycogen and glycogenolysis and gluconeogenic pathways, the liver produces free glucose from glycogen and gluconeogenic substrates, which is released into the circulation to serve as a fuel source for the organs and particularly, the brain [19]. If the blood contains insufficient amounts of glucose, only the liver [21] is able to produce ketone bodies, which are utilized as an additional energy source in the brain.

Nevertheless, both liver ketogenesis and gluconeogenesis are understudied areas (with the exception of a few studies) in terms of their impact on the adequate supply of the brain with energy substrates and clinical manifestations of HE [20]. Moreover, based on scattered and conflicting data from patients with fulminant liver failure or chronic liver diseases [22,23,24], it is impossible to assess how the reduction in ketone body and glucose output in the liver may be related to brain pathology. It should be noted that HE studies on animal models also show contradictory results. After portacaval shunt in rats, plasma levels of acetoacetate and β-hydroxybutyrate were reduced [25], or not significantly different from those in the sham-operated group [26]. In the animal model of hyperammonemia initiated by a long term diet containing ammonia, the level of ketone bodies in the blood rose significantly, hence, synthesis and export of ketone bodies by the liver in the hyperammonemic rats were supposed to increase [27].

In our studies performed in rat models of “pure” hyperammonemia produced by infusion of ammonium salts, we observed a significant decrease in the levels of both acetoacetate and β-hydroxybutyrate in the liver and blood of feeding animals, which suggested that hepatic ketogenesis can be inhibited immediately after increasing ammonia levels in the liver [28].

Given the importance of hepatic metabolic activity for providing fuel to the brain and an effective understanding of how perturbations in the metabolic capacity of liver cells may be related to brain pathology in HE, we therefore focused on hepatic gluconeogenesis and ketogenesis in animal models, investigating acute ammonia intoxication in which the concentration of ammonia in the blood quickly increases to the level found in idiopathic hyperammonemia following lung transplantation [29] and many other human pathologies [30].

Since these metabolic processes are energy-dependent and closely related to mitochondrial function and fatty acid oxidation [31], we sought to study the effect of acute hyperammonemia on the hepatic mitochondrial function, including oxidative phosphorylation, fatty acid oxidative capacity, and how fluctuation of glucose and ketone body levels in the blood can affect brain bioenergetics and coma development. This research will advance understanding of the role of liver mitochondria in brain energy metabolism.

## 2. Experimental Section

### 2.1. Experimental Design

This study was conducted in accordance with the ethical principles formulated in the Helsinki Declaration for care and use of laboratory animals and the Regulations of the European Science Association (revised in the instruction 86/609/EC and formulated in the Order of the Ministry of Health of the Russian Federation of 19.06.2003 № 267 “Regulations in laboratory practices”).

### 2.2. Animals

Groups of eight male Wistar rats weighing 200–220 g were used. The animals were housed in a vivarium, four per split-level cage (40 × 30 × 20 cm) at room temperature under a natural light regime. Well-fed animals were fed a standard diet. For hepatic gluconeogenic and ketogenic process activation during fasting, some animals were starved twenty-four hours before the beginning of the experiment.

The ammonia group was injected intraperitoneally with a lethal dose of ammonia acetate of 12 mmol/kg. As observed previously [32], the animals exhibited hyperventilation, clonic convulsions, and coma soon after ammonia injection and died in 15 ± 2 min. In this study, the rats were decapitated immediately after the first seizure, which usually occurred 10 min after ammonia injection. In some experiments, the decapitation procedure was carried out at 5 or 15 min after ammonia loading. To obtain the values for the zero time point, rats were injected with ammonium acetate and decapitated immediately.

Control group animals were decapitated at different time points after injection of saline or sodium acetate.

### 2.3. Preparative and Analytical Methods

Blood was drawn from the retro-orbital plexus into citrate-treated tubes. Plasma was deproteinized with 6% HClO_4_ and 40% ethanol and neutralized with KOH to pH 6.

Ammonia was estimated by a microfluorimetric method as described by Kosenko et al., 2008 [33].

Glucose, ketone bodies, oxaloacetate, and adenine nucleotides were determined by fluorometric enzymatic methods, as described earlier [28].

BioVision fluorometric assay kits (Catalog # K646-100), based on glucoamylase that hydrolyzes glycogen to glucose, were used to determine glycogen levels in freeze-clamped liver and brain samples.

Glycerol concentration was measured by an assay kit (Sigma, CN FG0100) according to the manufacturer’s instructions, using the procedure with a coupled enzyme assay.

Free fatty acids (FFA) were determined by the Free Fatty Acid Quantitation Kit (Sigma, CN MAK044) using a coupled enzyme assay according to the manufacturer’s instructions.

### 2.4. Freeze-Clamped Liver and Brain Neocortex

After decapitation, tissue specimens from liver and cortex tissues were removed and freeze-clamped as soon as possible in liquid nitrogen, and then, ground in a mortar into a fine powder. Three milliliters of 6% cold (−20 °C) perchloric acid/40% ethanol mixture were added to 0.3 g of tissue powder and were extracted for 5 min at −5 °C. Analytical techniques for preparation of liver and brain tissue extracts were similar to those used for blood plasma extraction.

### 2.5. Isolation of Liver Mitochondria Using a Self-Generated Percoll Gradient

Mitochondria were isolated from the remainder of the liver (after freeze clamping) by a combination of differential and self-generated Percoll gradient centrifugation, according to a protocol developed by Graham [34].

Mitochondrial protein was determined by the Lowry method (Lowry et al. 1951) with bovine serum albumin as standard [35].

### 2.6. Mitochondrial Purity Assessment

A quantitative estimate of mitochondrial fraction purity was made using marker enzymes: lactate dehydrogenase (contamination of cytosol), glucose-6-phosphatase (microsomal marker), urate oxidase (peroxisome marker), and 5’-nucleotidase (plasma membrane marker). The enzyme activities were determined by previously described methods [36].

β-Galactosidase, used for detection of lysosome admixture, was determined according to the method described by Graham (1993) [37].

Contamination of the mitochondrial samples with peroxisomes, microsomes, cytoplasm, plasma membranes, and lysosomes was 0.29 ± 0.075%, 0.43 ± 0.07%, 0.15 ± 0.04%, 0.25 ± 0.09%, and 0.34 ± 0.01%, respectively.

The yield of succinate dehydrogenase was more than 90% of total activity (390 ± 12.3 and 19.1 ± 2.1 nmol/min x mg protein in mitochondria and homogenate, respectively) consistent with Graham’s findings [38].

Together with a relatively low contamination of the mitochondria with other cellular organelles, the results indicate a high-purity mitochondrial fraction.

### 2.7. Study of Mitochondrial Respiration

Parameters of oxidative phosphorylation were recorded polarographically with a Clark-type electrode using the analytical techniques described earlier [36].

### 2.8. Determination of Pyruvate Carboxylase Activity (EC 6.4.1.1)

Liver mitochondria were disrupted by osmotic shock in 2 volumes of 5 mM potassium phosphate buffer (pH 7.0) containing 0.1 mM EGTA-K^+^ and 0.1 mM dithiothreitol (DTT), 0.1 mM phenylmethylsulfonyl fluoride (PMSF), 0.15 µM aprotinin, 1.5 µM, pepstatin, 10 µM leupeptin and by three subsequent cycles of freezing–thawing. After the final thawing step, the sample was centrifuged at 20,000g for 10 min at +4 °C, and the resulting supernatant was used to determine enzyme activity by oxidation of NADH at 340 nm using malate dehydrogenase (MDH) as a coupling enzyme [39].

### 2.9. Determination of Phosphoenolpyruvate Carboxykinase Activity (EC 4.1.1.32)

Liver phosphoenolpyruvate carboxykinase (PEPCK) activity was determined in a cytosolic fraction obtained from the postmitochondrial supernatant after centrifugation at 100,000× g for 30 min at +4 °C. An assay was performed by coupling the PEPCK with MDH and by NADH oxidation at 340 nm [40].

### 2.10. Microsome Isolation and Glucose-6-Phosphatase Activity (EC 3.1.3.9)

The microsomal fraction was obtained with a standard procedure of differential centrifugation without further purification. To remove the lysosome, the postmitochondrial supernatant was centrifuged at 20,000× g for 20 min at 4 °C and the resulting supernatant was centrifuged at 100,000× g for 30 min at 4 °C. The microsomal pellet was washed once with 20 mL of incubation medium. The final pellet was resuspended in the medium, containing 0.225 M sucrose, 10 mM HEPES, pH 7.4 (SH solution), 0.1 mM DTT, 0.1 mM PMSF, 0.15 µM aprotinin, 1.5 µM, pepstatin, and 10 µM leupeptin at a concentration of 10 mg/mL, and was used immediately to determine microsomal intactness by estimating the latency of “low Km” mannose-6-phosphatase activities in untreated microsomes [41]. For the glucose-6-phosphatase (G-6-Pase) assay, microsomes (50 µg of protein) were disrupted in SH solution containing 0.1% Triton X-100 at 0° for 20 min under constant gentle stirring. Enzyme activity was measured by following inorganic phosphate liberated at 700 nm using the method described by Baginski, 1974 [42].

### 2.11. Activity of Enzymes of Ketone Body Synthesis in the Liver

For the enzymes of the 3-Hydroxy-3-Methylglutaryl-CoA (HMG-CoA) pathway, the liver mitochondria were disrupted, and enzymes were solubilized using a technique similar to those used for pyruvate carboxylase determination.

### 2.12. Acetoacetyl-CoA Thiolase (EC 2.3.1.9)

The enzyme was determined using the method described by Williamson, 1968 [43] with minor modifications. The standard reaction mixture contained 50 mM Tris-HCl (pH 8.1), 5 mM MgCl_2_, 60 µM CoA, 10 µM acetoacetyl-CoA, 0.5 mM DTT, and 40 mM KCl. After pre-equilibration at 30 °C, the reaction was initiated by adding 20–40 µg of the sample in a total volume of 0.5 mL and the change in absorption at 313 nm was monitored for 10 min.

### 2.13. HMG-CoA Synthase (E.C.4. l.3.5)

Enzyme activity was estimated spectrophotometrically by measuring the initial rate of acetyl-CoA-dependent disappearance of acetoacetyl-CoA at 300 nm according to Quant et al., 1989 [44], with minor modifications. The assay system contained 50 mM Tris/HCl, pH 8.2, 0.1 mM DTT, 5 mM acetyl phosphate, 10 µM acetoacetyl-CoA, 100 µM acetyl-CoA, 20–50 µg of sample protein and 10 units of phosphate acetyltransferase (EC 2.3.1.8). HMG-CoA synthase activity was measured as the difference in the rate before and after acetyl-CoA addition. As mitochondrial HMG-CoA synthase is inhibited by Mg^2+^ [45], the assay was performed in the absence of MgCl_2_, where acetoacetyl-CoA has an ε300 = 3.6 × 10 ^3^ M ^-1^cm ^-1^ [46].

### 2.14. HMG-CoA Lyase (E.C.4.1.3.4)

HMG-CoA lyase activity was measured by a slightly modified Wanders procedure [47]. In short, 50–100 µg of the sample were incubated at 30 °C in 1 mL of medium containing 50 mM glycylglycine (pH 8.2), 5 mM MgCl_2_, 0.1 mM DTT, and 2 mM D,L-3-hydroxy-3-methylglutaryl-CoA. At the time point of 30 min, the reaction was stopped by perchloric acid precipitation (0.3 mL, 13%/40% ethanol) and the extracts were prepared using a technique similar to those used for the liver tissues, then, immediately used for fluorometric determination of acetoacetate [48].

### 2.15. 3-Hydroxybutyrate Dehydrogenase (E.C.1.1.1.30)

3-hydroxybutyrate dehydrogenase (HBDH) activity was measured in submitochondrial particles [49] by the method described earlier [50].

### 2.16. Analysis of Ketogenesis in Mitochondria

The rate of liver ketogenesis was measured in the isolated liver mitochondria using the technique of Takeyama et al., 1990 [51], and the end products of ketogenesis acetoacetate and β-hydroxybutyrate were determined by fluorometric enzymatic methods as described earlier [28].

### 2.17. Statistical Analysis

The results were expressed as mean ± SEM (standard error of the mean). Statistical processing of the results was performed using the program Prizm 5.0 for Windows (GraphPad Software, San Diego, CA, USA). The normality of the distribution of variables was confirmed by the Kolmogorov–Smirnov test. Pairwise comparisons were carried out using the Student’s *t*-test, and multiple comparisons were performed using the ANOVA and Bonferroni corrections.

## 3. Results

### 3.1. Metabolic Changes in the Liver and Blood during Starvation

**Figure 1:**
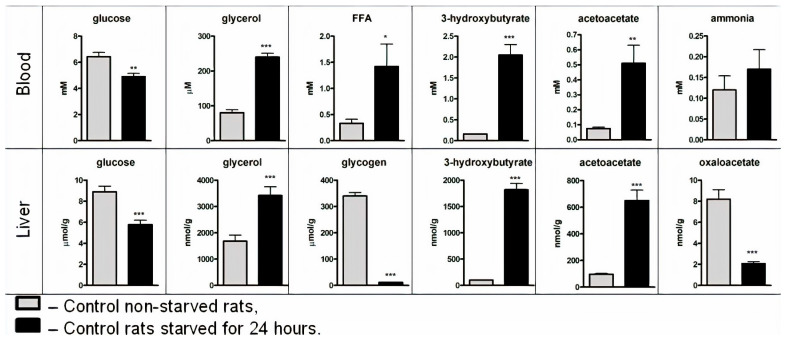
Metabolites in the blood and liver of non-starved rats and rats starved for 24 h. Values are the means ± SEM (*n* = 8). Significant differences in the values in “Control” are estimated by the Student’s *t*-test: * *p* < 0.05, ** *p* < 0.01, *** *p* < 0.001.

To establish how the presence of ammonia modifies the homeostatic function of the liver associated with maintaining glucose and ketone bodies levels in the blood under fasting conditions, we need to study the metabolic characteristics of starved animals without ammonia. We first examined concentrations of key metabolites in the blood and liver of control non-starved and starved animals (Figure 1).

As shown, 24 h after starvation, glycogen in the liver was significantly depleted (Figure 1). Simultaneously, there was a multifold increase in the concentration of glycerol, acetoacetate, and 3-hydroxybutyrate in the liver and blood; ammonia levels remained constant; FFA in the blood increased; glucose levels became lower, but still remained within the reference values. These results are consistent with the well-documented increase in hepatic gluconeogenesis, ureagenesis, and ketogenesis in starved rats.

### 3.2. Effect of Acute Ammonia Intoxication on Levels of Liver and Blood Glucose, Ketone Bodies, FFA, and Ammonia

We next examined whether ammonia could affect glucose and ketone body levels during fasting-induced hepatic gluconeogenesis and ketogenesis.

**Figure 2:**
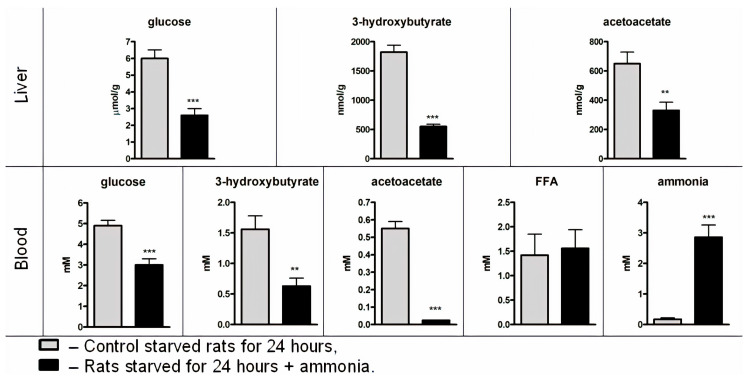
Metabolites in the blood and liver of starved rats 10 min after injection of ammonium acetate. Values are the means ± SEM (n = 8). Significant differences in the values in the group “24 h starvation” are estimated by the Student’s *t*-test: ** *p* < 0.01, *** *p* < 0.001.

As shown in Figure 1, the concentration of ammonia in the blood of control starved rats was 0.170 ± 0.047 mM and increased to 2.86 ± 0.4 mM (Figure 2) 10 min after ammonia injection, which was close to the level observed in non-starved rats with hyperammonemia [52] and in individuals with different pathologies [29,30].

Hepatic glucose, 3-hydroxybutyrate, and acetoacetate levels rapidly decreased, and 10 min after, the ammonia injection showed a 57%, 70%, and 49% decline, respectively, compared to control (Figure 2), suggesting that production of these metabolites in the liver was inhibited and a decrease in their blood levels was expected. Indeed, although the concentration of FFA in the plasma of rats with hyperammonemia was accessible to the hepatic mitochondrial β-oxidation and to the maintenance of ketogenesis and gluconeogenesis, 3-hydroxybutyrate concentration decreased by 60%, acetoacetate virtually disappeared from the blood and the blood glucose level decreased to 61% of control (Figure 2). These results imply that ammonia could disturb either the oxidative function of mitochondria or the metabolic pathway of HMG-CoA.

In order to identify the possible role of liver mitochondria in disrupting the ketogenesis and gluconeogenesis processes, we next studied the effect of acute hyperammonemia on the hepatic mitochondrial function, including oxidative phosphorylation and fatty acid oxidative capacity, first evaluating the intactness and purity of isolated mitochondria.

### 3.3. Intactness and Coupling Efficiency of Isolated Mitochondria

The intactness of isolated mitochondria was controlled by measuring the parameters of oxidative phosphorylation. The V_3_, V_4_, and V_unc_ rates during oxidation of glutamate + malate as respiratory substrates were 72.74 ± 1.91, 10.44 ± 0.84, and 97.2 ± 3.83 ng atoms O/min per 1 mg of mitochondrial protein, respectively. The respiratory control index (RCI) and ADP/O ratio were 7.2 ± 0.64 and 2.87 ± 0.21, respectively (see Table 1). The RCI values upon oxidation of other substrates—pyruvate + malate, succinate, oleate + carnitine, palmitoylcarnitine, and octanoate—were close to the theoretical values (Table 1) and indicated intactness and coupling of mitochondria at all three points of oxidative phosphorylation.

### 3.4. Oxidative Phosphorylation in Hyperammonemia

The ammonia concentration in liver mitochondria as calculated per intramitochondrial water [53] was 15 ± 4.23 mM in control rats and 95.7 ± 7.5 mM in animals with hyperammonemia.

Upon oxidation of pyruvate + malate in liver mitochondria of hyperammonemic rats, V_3_, RCI and ADP/O ratio were significantly lower (41%, 36%, and 16%, respectively) than the corresponding values for control samples (Table 1). The V_4_ and V_unc_ rates did not differ statistically across the two groups. With glutamate + malate or succinate in the presence of rotenone, the V_3_ rate also significantly decreased in hyperammonemia by 36% and 38%, respectively, but with only glutamate + malate, RCI fell slightly (22%), while the ADP/O ratio was essentially unchanged in the presence of both substrates in ammonia-treated rats. With the substrates of lipid nature palmitoylcarnitine, octanoate, and oleate + L-carnitine, the respiration rate, V_3_, in mitochondria from rats with hyperammonemia was markedly and equally inhibited (about 42%) compared to control. Upon oxidation of palmitoylcarnitine, octanoate, and oleate + L-carnitine, RCI in hyperammonemia significantly decreased by 55%, 50%, and 40%, respectively (Table 1).

These results show that the oxidative metabolism of both lipid and non-lipid substrates is disturbed during a multifold increase in ammonia concentration in the liver mitochondria. From this, we can infer that ammonia has a dramatic impact on mitochondrial oxidative phosphorylation, the main energy supplier in hepatocytes [31].

### 3.5. Adenine Nucleotides and NAD/NADH Ratio in Liver Mitochondria

To estimate the effect of ammonia on availability of the energy necessary to maintain homeostatic liver function, we measured total mitochondrial adenine nucleotide content, as well as the NAD/NADH ratio, which is known to regulate ketone body and glucose production [54].

**Figure 3:**
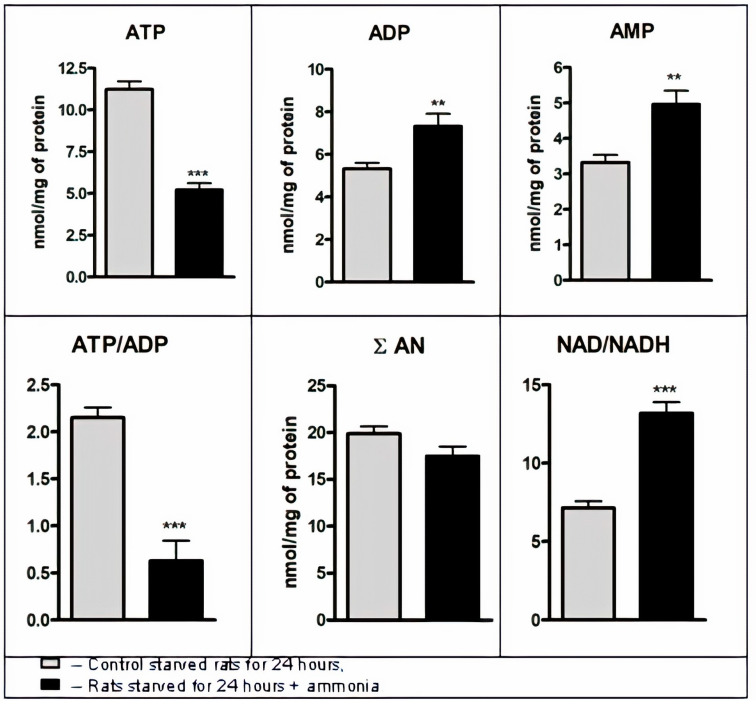
Distribution of adenine nucleotides and NAD/NADH ratio in liver mitochondria from control starved rats and starved rats treated with ammonia. Adenine nucleotide content in mitochondria was expressed as nmol/mg of protein. Mitochondrial free [NAD^+^]/[NADH] ratio was calculated from the components of the glutamic dehydrogenase ([NAD^+^]/[NADH] = [a-ketoglutarate][NH_4_^+^]/[glutamate], where the equilibrium constant was 3.87 [55]. Values are the means ± SEM (*n* = 8). Significant differences in the values in the group “Control” are estimated by the Student’s *t*-test: ** *p* < 0.01, *** *p* < 0.001.

The results show that 10 min after injection of ammonia into the starved animals, the ATP content in mitochondria fell by 54% compared to control, while inversely, ADP increased by 38%, and AMP by 49% (Figure 3). The total content of adenine nucleotides (AN) changed insignificantly, while the ATP/ADP ratio dramatically decreased by 71% during this time interval. This indicates that the energy potential of adenine nucleotides in liver mitochondria is decreased by the action of ammonia, but subsequent AN degradation occurs relatively slowly.

At the same time, the redox state of pyridine nucleotides in the mitochondrial matrix changed rapidly; having abruptly dropped upon starvation of control rats from 20 to 7 (data not shown), the NAD/NADH ratio rose immediately up to 13.2 (by 85% higher than the initial value) 10 min after injection of ammonia (Figure 3).

The findings agree with earlier reported results [56] obtained using liver mitochondria from fed rats with hyperammonemia. They suggest that the more oxidized mitochondrial redox state, together with the diminished ATP content and ATP/ADP ratio would result in cessation of energy production followed by a decrease in the activity of pyruvate carboxylase, the first ATP-dependent mitochondrial enzyme that triggers gluconeogenesis [54].

### 3.6. Effect of Ammonia on Pyruvate Carboxylase, Phosphoenolpyruvate Carboxykinase, and Glucose-6-Phosphatase Activity

**Figure 4:**
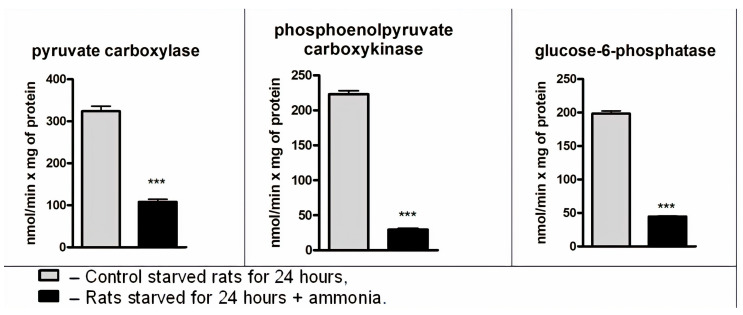
Effect of ammonia on the activity of pyruvate carboxylase (PC), Phosphoenolpyruvate Carboxykinase (PEPCK) and Glucose-6-Phosphatase (G6Pase) in the liver of starved rats. The activities of PC, PEPCK and G6P-ase in the liver mitochondria, cytosol, and microsomes are expressed in nmol/min per 1 mg of protein of the corresponding fraction. Values are the means ± SEM (*n* = 8). The significant differences in the values in the group “24 h starvation” are estimated by the Student’s *t*-test: *** *p* < 0.001.

We next evaluated whether the ammonia would affect the activity of mitochondrial pyruvate carboxylase (PC) and other key rate-limiting gluconeogenic enzymes located outside the mitochondria. Figure 4 shows that 10 min after injection of ammonia, an abrupt decrease in the activities of PC (67%), PEPCK (87%), and G6Pase (78%) occurred in the liver mitochondria, cytosol, and microsomes, respectively.

These findings show that in starved animals characterized by enhanced hepatic gluconeogenesis, by which normal blood glucose concentration is maintained for at least 24 h upon starvation, the accumulation of ammonia in different liver cell compartments causes a rapid and dramatic decrease in the activities of all key rate-limiting gluconeogenic enzymes; this results in a decline in basal rates of hepatic glucose production and fasting plasma glucose concentrations (Figure 2).

### 3.7. Effect of Ammonia on Enzyme Activities of the HMG-CoA Pathway and Acetoacetate Production in the Liver Mitochondria of Starved Rats

**Figure 5:**
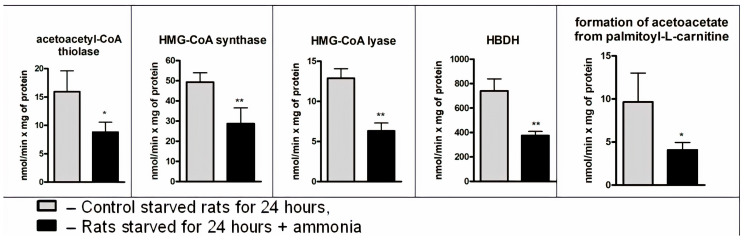
Activities of the enzymes of the HMG-CoA pathway and rate of acetoacetate formation in the mitochondria of control and hyperammonemic rats. The activity of enzymes and the rate of acetoacetate formation are expressed in nmol/min per 1 mg of mitochondrial protein. Values are the means ± SEM (*n* = 8). Significant differences in the values in the group “24 h starvation” are estimated by the Student’s *t*-test: * *p* < 0.05; ** *p* < 0.01.

In some pathological conditions, there is a correlation between gluconeogenesis inhibition and reduced total blood ketone body concentrations, suggesting that inhibition of gluconeogenesis may decrease ketogenesis [57], so we next examined whether hepatic ketogenic capacity in starved animals would actually be altered by ammonia. As can be seen in Figure 5, its injection produced a significant decrease in the specific activities of acetoacetyl-CoA thiolase (45%), HMG-CoA synthase (42%), HMG-CoA lyase (51%), and HBDH (49%). Additionally, the acetoacetate formation rate decreased twice as much as control (Figure 5), simultaneously with its complete disappearance from the blood (Figure 2).

To assess how fluctuations in glucose and ketone bodies in the blood could affect brain bioenergetics and coma development, we studied changes in concentrations of ketone bodies and glucose in the liver, blood, and brain after the first 15 min of loading with ammonia.

**Figure 6:**
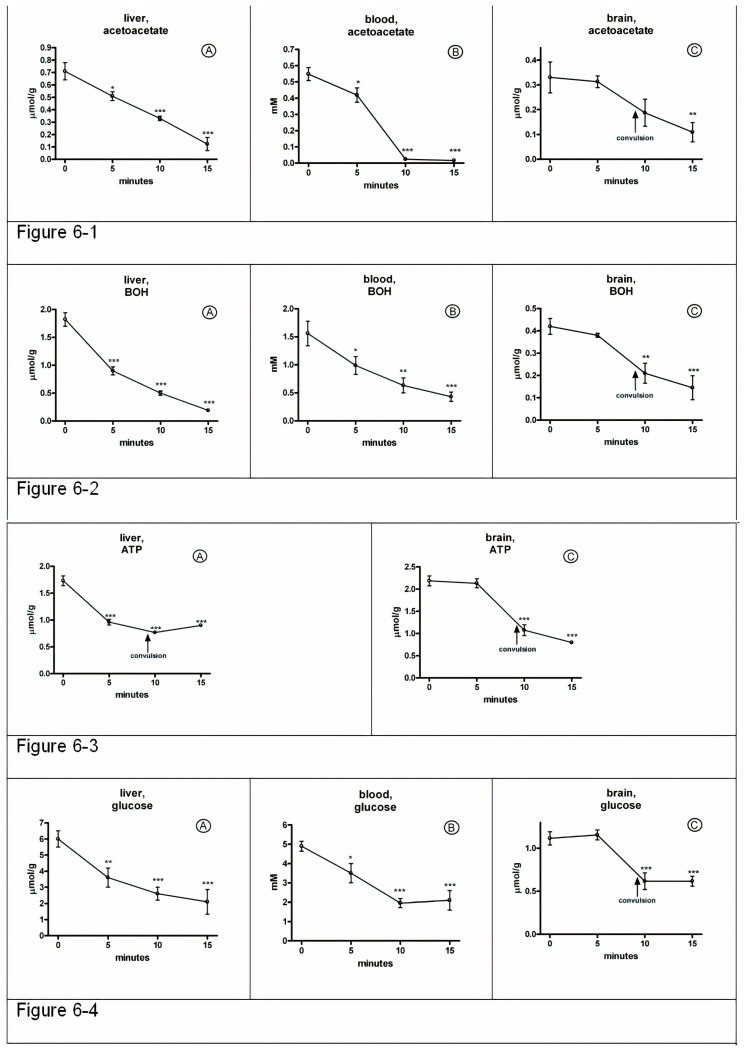
Time course of changes in acetoacetate, 3-hydroxybutyrate, ATP, and glucose in the liver, blood, and brain in acute ammonia intoxication. Values are the means ± SEM (*n* = 8). Significant differences between the values with respect to the time point of 0 min were estimated by ANOVA analysis and corrected with Bonferroni: * *p* < 0.05, ** *p* < 0.01, *** *p* < 0.001.

Concentrations of acetoacetate and 3-hydroxybutyrate decreased within the first 5 min by 28% and 50% in the liver, 24% and 37% in the blood (Figure 6-1A and Figure 6-2A; Figure 6-1B and Figure 6-2B), with no change in the brain (Figure 6-1C and Figure 6-2C). Within the next 5 min (10 min after injection), the concentration of ketone bodies continued to decrease in the liver, blood, and finally, in the brain (Figure 6-1C and Figure 6-2C).

Similar changes in the concentration of ATP (Figure 6-3A,C), the total content of adenine nucleotides, as well as the energy charge of adenylate (data not shown) and glucose (Figure 6-4A,B,C) occurred in the brain; concentrations of all metabolites remained unchanged within the first 5 min after injection of ammonia, but sharply decreased just before seizures (by the time point of 10 min), which suggests a time-dependent development of metabolic events—first in the liver, then in the blood and, finally, in the brain.

Thus, these results suggest that there is a relationship between the inhibition of hepatic gluconeogenesis, and ketogenesis, resulting in decreased availability of cerebral glucose and ketone bodies to the brain and bioenergetic collapse of the brain, accompanied by a cascade of pathological reactions [58], leading to irreversible changes in the brain and development of coma as a terminal event in the toxicity of ammonia.

## 4. Discussion

Although the relationship between liver damage and neuropathology has been known for over a century, the genesis of encephalopathy under impaired liver function is not yet completely resolved.

Nonetheless, more recent studies have provided evidence for a paramount role of the liver, reporting the main cause of HE as ammonia accumulation in the blood and brain due to impaired detoxification function of the liver or by portosystemic shunt [3].

In fact, the liver performs a myriad of functions, and in addition to filtering toxins, plays a unique role in the regulation of blood glucose homeostasis, crucial for normal cerebral function, since the brain neither synthesizes nor stores the required amount of glucose [59]. As a result, unstable blood glucose and oxygen delivery to the brain, for even a short period, causes brain damage, while chronic deficiency in these substrates leads to irreversible brain injury, thereby triggering cognitive impairment, coma development, and death [20]. Additionally, when glucose is not readily available, the liver can produce ketone bodies, which are used by the brain as an alternative energy source.

We previously showed that acute hyperammonemia leads to rapid and significant decrease in both acetoacetate and β-hydroxybutyrate levels in the liver and blood of non-starved rats. This suggests that hepatic ketogenesis can be inhibited immediately after increasing ammonia levels in the liver [60], highlighting the role of deterioration of homeostatic liver functions in the occurrence of a brain energy crisis [20].

In light of the notable lack of studies evaluating the effect of ketogenesis and gluconeogenesis in the liver on brain energy metabolism, our study focused on hepatic gluconeogenesis and ketogenesis, including oxidative phosphorylation in mitochondria and the oxidative ability of fatty acids in animal models of acute ammonia intoxication. In this model, ammonia concentrations in the blood quickly increase to the level found in idiopathic hyperammonemia associated with many human pathologies [29,30] and lead to rapid encephalopathy, which underlines the importance of our study.

We revealed that oxidative phosphorylation in rat liver mitochondria was rapidly and progressively suppressed by high mitochondrial ammonia levels.

While ammonium acetate 10 min after administration to the starved rats induced a decrease in state 3 rate and RCI, state 4 respiration remained unchanged during oxidation of lipid and non-lipid substrates (Table 1). These effects of ammonia were reproduced in liver [61] and non-synaptic brain mitochondria [9] of non-starved rats and according to the known action of the uncouplers [62], it was suggested that ammonia seems to have an inhibitory but not uncoupling effect on state 3 respiration and on the electron transfer along the respiratory chain. Finally, deficiency in cytochrome oxidase [63] and degradation of cytochrome-C in liver mitochondria of hyperammonemic rats [64] lead to the same type of oxidative phosphorylation disorders. These findings could explain why ammonium-induced dysfunction of liver mitochondria leads to a sharp drop in the ATP levels in these organelles.

Another consequence of ammonia accumulation is the NO-dependent accelerated formation of superoxide radicals and a simultaneous decrease in the activity of antioxidant enzymes in liver mitochondria [13]. We therefore hypothesized that NO-dependent oxidative stress would be involved in the mitochondrial toxicity of ammonia.

Indeed, we found that despite increased blood glucagon and intramitochondrial levels of acetyl-CoA (essential activators of PC) [60] in starved rats, ammonia loading enhanced the NAD/NADH ratio (Figure 3); ADP level in mitochondria, a potent PC inhibitor [54], together with ATP deficiency led to a significant decrease in PC activity (65%, p < 0.001).

Since there is a causal relationship between PC activity and the whole gluconeogenesis pathway [65], a decrease in activity of other gluconeogenic rate-limiting enzymes was expected. Further studies (Figure 4) showed progressive decrease in liver PEPCK (87%) and G-6-Pase (76%) activity 10 min after ammonia injection.

Thus, we have shown for the first time that in acute hyperammonemia, hepatic gluconeogenesis is inhibited at the level of at least three key enzymes. Accordingly, liver and blood glucose levels began to decline within 5 min (42% and 29%, respectively, compared to the zero time (Figure 6-4A,B)) after ammonia injection, but brain glucose (Figure 6-4C) and ATP levels (Figure 6-3C) were unchanged and behavior of the animals did not differ from control for the same time period. Later on, however, before the seizures, when blood glucose level fell below 2 mM, brain glucose and ATP concentration decreased by 45% and 52%, respectively, and no further significant changes were observed after repeated seizures and coma (Figure 6-4C, Figure 6-3C). Therefore, ammonia-induced acute hypoglycemia resulting from the gluconeogenesis inhibition may precede the onset of seizures and can lead to coma and culminate in neuronal death.

Whereas the levels of ketone bodies in the liver and blood changed in a similar way to glucose levels after ammonia administration (Figure 6-1B, Figure 6-2B), suppression of ketogenesis was predictable.

Indeed, ammonium intoxication caused a significant (approximately twofold) decrease in activity of acetoacetyl-CoA thiolase, HMG-CoA synthase, HMG-CoA lyase, and HBDH (Figure 5). As a result, the acetoacetate formation rate significantly reduced in mitochondria of ammonia-treated animals (Figure 5). We found that the reversal of ketogenesis of starvation occurred due to ammonia within 5 min and became more pronounced just before the first seizure, with no change after the following seizures and coma (Figure 6).

Taken together, these findings indicate that high ammonia level in vivo has a dramatic effect on liver mitochondria as a prooxidant and redox stressor. More importantly, liver mitochondria dysfunction is an early and critical stage in development of ammonia-induced hepatotoxicity, preceding encephalopathy. Given the time-dependent link between abnormalities in the liver and the development of seizures, we hypothesize that HE is a state of the body, in which there is a discrepancy between demand in the brain for energy substrates produced by the liver and inability of the liver to generate these substrates. Our study, thus, provides a framework to further explore the relationship between hepatic dysfunction and progression of brain energy crisis in hepatic encephalopathy.

## Figures and Tables

**Table 1:** Parameters of oxidative phosphorylation in liver mitochondria of control starved animals and animals treated with ammonium acetate.

Substrate	V3	V4	Vu	RCI	ADP/O
**Control Animals Starved for 24 h**					
Pyruvate + malate	30.24 ± 1.099	10.11 ± 0.11	36.2 ± 2.23	2.99 ± 0.16	3.021 ± 0.13
Glutamate + malate	88.52 ± 4.37	12.24 ± 0.98	95.3 ± 4.54	7.12 ± 0.54	2.87 ± 0.21
Succinate + rotenone	117 ± 5.45	22.8 ± 0.87	141 ± 5.31	5.13 ± 0.36	1.64 ± 0.043
PalmitoyI-L-carnitine	45.16 ± 1.73	10.33 ± 1.81	81.04 ± 5.33	4.37 ± 0.29	1.83 ± 0.44
Octanoate Na	24.2 ± 0.9	4.70 ± 0.11	53.7 ± 3.41	5.15 ± 0.28	2.02 ± 0.06
Oleic acid +L- carnitine	18.5 ± 0.61	6.17 ± 0.23	54.4 ± 3.36	3.0 ± 0.17	2.07 ± 0.19
**Ammonia-Treated Animals Starved for 24 h**					
Pyruvate + malate	17.84 ± 2.43 ***	9.32 ± 1.13	33.96 ± 2.63	1.91 ± 0.21 **	2.54 ± 0.09 *
Glutamate + malate	56.98 ± 3.61 ***	10.32 ± 2.12	96.2 ± 3.52	5.52 ± 0.27 *	2.46 ± 0.31
Succinate + rotenone	72.3 ± 1.33 ***	19.11 ± 3.33	134 ± 6.20	3.78 ± 0.53	1.77 ± 0.075
PalmitoyI-L-carnitine	23.9 ± 1.7 ***	12.1 ± 1	102 ± 11	1.98 ± 0.12 ***	2.45 ± 0.15 *
Na Octanoate	14 ± 0.52 ***	5.5 ± 0.29 *	49.8 ± 1.5	2.55 ± 0.14 ***	2.7 ± 0.2 **
Oleic acid + L- carnitine	10.6 ± 0.55 ***	7.1 ± 1.45	54.5 ± 7.5	1.8 ± 0.25 **	2.7 ± 0.11 **

V_3_, V_4_ and V_unc_ are the mitochondrial respiration rates in the presence of 200 μM ADP (state 3), in the absence of ADP (state 4), and in the presence of 0.5 μM carbonylcyanide-m-chlorophenylhydrazone (CCCP, state of respiration uncoupling), respectively, and are expressed as ng atom O/min per 1 mg of mitochondrial protein; RCI = V_3_/V_4_ is the respiratory control index. The ADP/O ratio reflects the efficiency of oxidative phosphorylation and is the ratio of the amount of ADP (in nanomoles) phosphorylated with the formation of ATP to the amount of oxygen (in nanogram atoms) utilized in state 3. The substrates were added at final concentrations of: 5 mM sodium pyruvate/2.5 mM potassium malate, 5 mM potassium glutamate/2.5 mM potassium malate, 10 mM potassium succinate/1 µM rotenone, 40 µM palmitoylcarnitine, 5 μM octanoate Na, and 20 μM oleic acid/2 mM carnitine. Values are the means ± SEM (n=8). Significant differences in the values in the group “24 h starvation” are estimated by the Student’s *t*-test: * *p* < 0.05, ** *p* < 0.01, *** *p* < 0.001.
